# Verification and rectification of cell type-specific splicing of a Seckel syndrome-associated ATR mutation using iPS cell model

**DOI:** 10.1038/s10038-019-0574-8

**Published:** 2019-03-08

**Authors:** Jose Ichisima, Naoya M. Suzuki, Bumpei Samata, Tomonari Awaya, Jun Takahashi, Masatoshi Hagiwara, Tatsutoshi Nakahata, Megumu K. Saito

**Affiliations:** 1 Department of Clinical Application, Center for iPS Cell Research and Application (CiRA), Kyoto University, Kyoto, 606-8507 Japan; 2 Department of Anatomy and Developmental Biology, Graduate School of Medicine, Kyoto University, Kyoto, 606-8501 Japan; 3 Department of Fundamental Cell Technology, Center for iPS Cell Research and Application (CiRA), Kyoto University, Kyoto, 606-8507 Japan

**Keywords:** Genetics of the nervous system, Induced pluripotent stem cells, Transcriptomics

## Abstract

Seckel syndrome (SS) is a rare spectrum of congenital severe microcephaly and dwarfism. One SS-causative gene is Ataxia Telangiectasia and Rad3-Related Protein (ATR), and ATR (c.2101 A>G) mutation causes skipping of exon 9, resulting in a hypomorphic ATR defect. This mutation is considered the cause of an impaired response to DNA replication stress, the main function of ATR, contributing to the pathogenesis of microcephaly. However, the precise behavior and impact of this splicing defect in human neural progenitor cells (NPCs) is unclear. To address this, we established induced pluripotent stem cells (iPSCs) from fibroblasts carrying the ATR mutation and an isogenic ATR-corrected counterpart iPSC clone. SS-patient-derived iPSCs (SS-iPSCs) exhibited cell type-specific splicing; exon 9 was dominantly skipped in fibroblasts and iPSC-derived NPCs, but it was included in undifferentiated iPSCs and definitive endodermal cells. SS-iPSC-derived NPCs (SS-NPCs) showed distinct expression profiles from ATR non-mutated NPCs with negative enrichment of neuronal genesis-related gene sets. In SS-NPCs, abnormal mitotic spindles occurred more frequently than in gene-corrected counterparts, and the alignment of NPCs in the surface of the neurospheres was perturbed. Finally, we tested several splicing-modifying compounds and found that TG003, a CLK1 inhibitor, could pharmacologically rescue the exon 9 skipping in SS-NPCs. Treatment with TG003 restored the ATR kinase activity in SS-NPCs and decreased the frequency of abnormal mitotic events. In conclusion, our iPSC model revealed a novel effect of the ATR mutation in mitotic processes of NPCs and NPC-specific missplicing, accompanied by the recovery of neuronal defects using a splicing rectifier.

## Introduction

Seckel syndrome (SS, OMIM: 210600) is a spectrum of congenital disorders that mainly exhibit intrauterine growth defects resulting in severe microcephaly and dwarfism [1]. So far, 10 genes have been identified as responsible for the onset of SS, and all of them show an autosomal recessive trait [2]. SS is a major type of microcephalic primordial dwarfism, and SS-related genes are associated with DNA damage response genes [3, 4] or centriole biogenesis genes [5, 6]. The first mutation found in these genes was in the *Ataxia Telangiectasia and Rad3-Related Protein (ATR)* gene in two Pakistani families [3, 7]. Since the fibroblast cell line of patients (ATR-SS) demonstrates a defective DNA replication stress response, the mutations in ATR and other DNA damage response genes that cause microcephaly are regarded to impair the response to DNA replication stress in neural progenitor cells (NPCs) [8, 9]. However, due to the limited availability of patient-derived NPCs, the precise pathogenic impact of these genes on human NPCs remains to be elucidated.

Five *ATR* mutations associated with SS have been reported to create splicing defects, and the first identified *ATR* mutation (c.2101 A>G), which locates in exon 9, induces aberrant splicing of this exon [3, 10–12]. Although the mutation is synonymous, homozygous mutations can reduce the translation of ATR protein due to premature termination of the mRNA [3]. Since the skipping of exon 9 occurs in a partial manner, thus keeping a small proportion of the correct full length (FL) isoform, this mutation is classified as hypomorphic. Because the impairment of neural development usually accompanies microcephalic disorders [1], studies that dissected the pathological mechanism of SS have mainly focused on the biology of NPCs. For this purpose, several reports have established an *ATR* (c.2101 A>G) mutation-specific model with humanized mouse alleles [13]. With this model, however, characteristics besides patient symptoms, such as ophthalmological abnormalities, were observed [14]. Other difficulties in studying the disease are the technical challenges in maintaining ATR knocked-out cells in vitro [15] and in differentiating them into neural lineage cells.

Recently, patient-derived induced pluripotent stem cells (iPSCs) have been applied to model the phenotypes of neurological disorders, including microcephalic syndromes [16], because they can produce a sufficient number of NPCs for experiments. Additionally, by establishing isogenic mutation-corrected control iPSCs using genome editing technology, the obtained phenotypes can be accurately evaluated. Here we report the first iPSC model of SS. We established iPSC clones from SS-patient-derived fibroblasts harboring 2101 A>G mutation (SS-iPSCs) and found cell type-specific aberrant splicing of ATR exon 9. We also identified novel neural phenotypes of SS. Finally, we succeeded in restoring the aberrant splicing in NPCs by a chemical compound. These findings provide novel insights into the pathophysiology of ATR-SS and a future potential pharmacological intervention to SS patients.

## Materials and methods

### Study approval

The study plan for recombinant DNA research was approved by the recombinant DNA experiments safety committee of Kyoto University. All methods were performed in accordance with the relevant guidelines and regulations. Animal studies were approved by the institutional review board.

### Cell lines

SS patient fibroblasts were obtained from the NIGMS Human Genetic Cell Repository at the Coriell Institute for Medical Research: GM18366. The human iPS cell line 409B2 was kindly provided by Dr. Shinya Yamanaka (Center for iPS Cell Research and Application, Kyoto University, Kyoto, Japan).

### Establishment and maintenance of iPSCs

Episomal vectors encoding reprogramming factors (OCT3/4, SOX2, KLF4, L-MYC, LIN28, and p53 shRNA) were transfected into fibroblasts on day 0 as described previously [17]. The transfected cells were reseeded onto feeder layers on day 7 and maintained in embryonic stem cell medium (ReproCELL, Yokohama, Japan). The iPSC clones were cultured on Laminin511-E8 fragment iMatrix-511 (Nippi, Tokyo, Japan)-coated tissue culture plates with StemFit AK02N medium (Ajinomoto, Tokyo, Japan) at 37 °C. For passaging, TrypLE Select (Thermo Fisher Scientific, Waltham, MA, USA) was used to dissociate cell colonies into single cells as reported previously [18].

### Genome editing of SS-iPSCs

The mutant ATR gene in SS-iPSCs was corrected by the CRISPR/Cas9 system. The guide RNA (gRNA) sequence was designed to recognize only the mutant allele of *ATR* (c.2101 A>G) (TCCGGGCTAGTTGTGTTAGT). iPSC clones cultured under feeder-free conditions were dissociated into single cells. Vectors expressing gRNA and Cas9 protein were then introduced with a NEPA21 electroporator (NEPAGENE, Chiba, Japan). Two days after transfection, cells were incubated with neomycin (50 μg/mL). The DNA of surviving colonies were extracted from the cells and genotyped by Sanger sequencing.

### Sanger sequencing

Sanger sequencing was performed with the BigDye Terminator v3.1 Cycle Sequencing Kit (Thermo Fisher Scientific), and the subsequent products were purified with the BigDye XTerminator Purification Kit (Thermo Fisher Scientific). The 3500xL sequencer (Thermo Fisher Scientific) was used for the analyses. Data were analyzed with CodonCode Aligner software (CodonCode Corporation, Centerville, MA, USA).

### RNA isolation, quantitative RT-PCR, and semiquantitative RT-PCR

Total RNA extraction from cells was performed using the RNeasy Mini Kit (QIAGEN, Germantown, MD, USA). One microgram of total RNA was used for the reverse transcription with PrimeScript RT Master Mix (TaKaRa, Shiga, Japan). Quantitative PCR was performed with SYBR Premix Ex TaqII (TaKaRa) in triplicate using the StepOnePlus Real-Time PCR system (Thermo Fisher Scientific). The expression of *glyceraldehyde-3-phosphate dehydrogenase (GAPDH)* was used as an endogenous control.

Semiquantitative RT-PCR was performed with KOD FX Neo (TOYOBO, Osaka, Japan) with the following cycle conditions: 94 °C for 2 min, followed by 35 cycles of denaturation at 98 °C for 10 s, annealing at 60 °C for 15 s, and elongation at 68 °C for 30 s, with a final 7-min incubation at 68 °C in a Veriti Thermal Cycler (Thermo Fisher Scientific). PCR products were pre-stained and separated by electrophoresis. Images were obtained with E-Graph E-Shot Imaging System (ATTO, Tokyo, Japan) and analyzed with ImageJ software (National Institutes of Health, Bethesda, MD, USA). We characterized the splicing patterns of *ATR* mRNA by RT-PCR with primers spanning exon 9. The ratios of exon inclusion/skipping were calculated as the amount of full-length transcript relative to the skipped transcript. The primer sets used for the quantitative PCR assay are described in Supplemental Table 1.

### Teratoma formation

The iPSCs recovered from one 6-cm dish were injected subcutaneously into NOG mice (Central Institute for Experimental Animals, Kawasaki, Japan). Tumors were dissected eight weeks after injection and fixed with PBS containing 4% paraformaldehyde. Paraffin-embedded tissues were sliced and stained with hematoxylin and eosin. Slides were examined using a BIOREVO BZ-9000 system (KEYENCE, Osaka, Japan).

### Protein isolation and western blot analysis

Cells were collected, suspended in RIPA buffer (Wako) supplemented with 1% protease inhibitor cocktail (Nacalai, Kyoto, Japan) and 1% phosphatase inhibitor cocktail (Nacalai), incubated on ice for 15 min and then centrifuged at 15,000 rpm for 15 min at 4 ℃. Each sample was separated on Bolt™ 4–12% Bis-Tris Plus Gels (Thermo Fisher Scientific), transferred to a PVDF membrane, and blocked with PVDF Blocking Reagent (TOYOBO). Blocked PVDF membranes were incubated with a primary antibody against Phospho-Chk1 (Ser345) Rabbit mAb (CST #2348, 1/1,000, Danvers, MA, USA), Chk1 Mouse mAb (CST #2360, 1/1,000), ATR Rabbit pAb (CST #2790, 1/1000) and β-Actin (13E5) Rabbit mAb (CST #5125, 1/2,500), followed by Anti-rabbit IgG, HRP-linked secondary antibody (CST #7074, 1/2,500) or Anti-mouse IgG, HRP-linked secondary antibody (CST #7076, 1/2,500), and then visualized using SuperSignal West Femto Maximum Sensitivity Substrate (Thermo Fisher Scientific). Images were acquired using a LAS4000 image analyzer (Fujifilm, Tokyo, Japan). Quantitative densitometry analysis was performed using ImageJ software.

### Immunocytochemistry and microscopy

Cells were fixed in 4% paraformaldehyde for 30 min at room temperature, permeabilized with 0.2% TritonX-100 PBS and incubated with 1xBlock Ace (DS PHARMA BIOMEDICAL, Osaka, Japan) containing 0.2% Tween-20 to prevent non-specific binding before overnight incubation with primary antibodies at 4 °C. The following day, secondary antibody incubations were performed for 1 h with the appropriate species-specific antiserum coupled to either FITC or Cy3 (Jackson ImmunoResearch #711-095-152, #711-165-152, #715-095-151, #715-165-151, 1/100, West Grove, PA, USA). After staining the nuclei with DAPI (Sigma-Aldrich, 1/1,000, St. Louis, MO, USA), the cells were mounted using Vectashield (Vector Laboratories, Burlingame, CA, USA) and imaged using BZ-X710 (KEYENCE). All antibodies were diluted in 1xBlock Ace. The following primary antibodies were used at the indicated dilution rates: PAX6 (BD Biosciences #561462, 1/100, Franklin Lakes, NJ, USA), NESTIN (MERCK #AB5922, 1/1,000, Darmstadt, Germany), SSEA-4 (R&D SYSTEMS #MAB1435, 1/100, Minneapolis, MN, USA), OCT-3/4 (R&D SYSTEMS #AF1759, 1/100), PERICENTRIN (Abcam #ab4448, 1/1,000, Cambridge, UK), and α-Tubulin (Sigma-Aldrich # T9026, 1/1,000) N-CADHERIN (Abcam #18203, 1/1000). For immunostaining of a definitive endodermal marker, Alexa Fluor 488-conjugated SOX17 (BD Biosciences #562205, 1/100) was used without secondary antibody.

### Neural differentiation

SFEBq-based differentiation was performed as previously described [19, 20]. iPSCs were dissociated into single cells and quickly re-aggregated in DFK 5% medium (DMEM/F-12 Ham medium (Sigma-Aldrich) containing KnockOut™ Serum Replacement (Thermo Fisher Scientific), NEAA (Thermo Fisher Scientific), 2-mercaptoethanol (Nacalai), GlutaMAX (Thermo Fisher Scientific), SB-431542, Dorsomorphin (Tocris, Minneapolis, MN, USA), and Y-27632 (Tocris)) (9000 cells/well) using a Nunclon Sphera Microplates 96U-Well Plate (Thermo Fisher Scientific). The medium was changed every 4 days until day 12, after that we changed to neurobasal medium supplemented with B27 supplement (Thermo Fisher Scientific) and GlutaMAX (Thermo Fisher Scientific) for further neural differentiation. Neurospheres were fixed in 4% paraformaldehyde for 30 min at room temperature followed by dehydration with 30% sucrose overnight and then cryosectioned at 20 μm with Leica CM1860 Cryostat (Leica Biosystems, Wetzlar, Germany).

### Mitotic spindle analysis

For the mitotic spindle analysis of NPCs, cell aggregates were dissociated on day 12 with Accumax (Innovative Cell Technologies, San Diego, CA, USA) and replated onto Corning® Matrigel® Growth Factor Reduced (GFR) Basement Membrane Matrix-coated dishes in DFK 5% medium. After 2 days, cells were immunostained on the dish. Mitotic spindles were classified into four mitotic phenotype classes manually as described previously [21]. In brief, normal (bipolar) and monopolar mitotic spindles were defined as containing two poles with either two or one centrosome present, respectively. When the spindle microtubules or DNA were not aligned properly, or if the centrosomes were detached, the cells were defined as unbalanced mitotic spindles. Multipolar mitotic spindles were defined as containing more than two centrosomes. Images were acquired and analyzed with BZ-X710 Analyzer (KEYENCE).

### Definitive endodermal cell induction

To differentiate iPSCs into definitive endodermal cells, we utilized a previously described protocol [22]. iPSCs were dissociated, resuspended with RPMI1640 (Sigma-Aldrich) medium containing 1 × B27 supplement, 100 ng/mL rhActivin A (R&D SYSTEMS), 1 μM CHIR99021 (Sigma-Aldrich), and 10 μM Y-27632 and seeded on culture dishes coated with Matrigel (Corning, Corning, NY, USA) at a density of 1 × 105 cells/cm^2^. On the next day, Y27632 was removed from the medium, and 0.5 mM NaB (FUJIFILM Wako Pure Chemical Corporation, Osaka, Japan) was added. Definitive endodermal cells were harvested by sorting with PE Mouse Anti-Human CD184 (BD Biosciences #555974) at day 5 using BD FACSAria II (BD Biosciences).

### Hematopoietic progenitor cell differentiation

Hematopoietic progenitor cells (HPCs) were generated as previously described [23]. Briefly, iPSCs were dissociated into single cells and quickly re-aggregated in Essential 8 Medium (Thermo Fisher Scientific) containing 80 ng/ml BMP4 (R&D SYSTEMS), 80 ng/ml VEGF (R&D SYSTEMS), 2 μM CHIR99021 and 30 μM Y-27632) (9000 cells/well) using a Nunclon Sphera Microplates 96U-Well Plate. Embryoid bodies were cultured at 37 °C, 5% CO_2_ and 5% O_2_ during differentiation. On day 2, the medium was removed, and Essential 6 Medium (Thermo Fisher Scientific) containing 80 ng/ml VEGF, 25 ng/ml bFGF (R&D SYSTEMS), 50 ng/ml SCF (R&D SYSTEMS) and 2 μM SB431542 (Tocris) was added. On day 4, the medium was replaced with Stemline® II Hematopoietic Stem Cell Expansion Medium (Sigma-Aldrich) containing 50 ng/ml SCF (R&D SYSTEMS), 20 ng/ml TPO (R&D SYSTEMS), 40 ng/ml VEGF and 50 ng/ml IL-3 (R&D SYSTEMS). HPCs were harvested by sorting with PE Mouse Anti-Human CD34 (BECKMAN COULTER, Brea, CA, USA #A07776) and FITC Mouse Anti-Human CD43 (Thermo Fisher Scientific #11-0439-42) at day 12 using BD FACSAria II (BD Biosciences).

### Validation of replication stress response

For the replication stress response assay on NPCs, the cell aggregates were dissociated on day 12 with Accumax (Innovative Cell Technologies) and replated onto Corning® Matrigel® Growth Factor Reduced (GFR) Basement Membrane Matrix-coated dishes in DFK 5% medium. NPCs were treated with HU (2 mM, 4 h), then harvested, and proteins were isolated as described above.

### Treatment of SS-NPCs with SR protein modifiers

Cell aggregates were dissociated on day 12 with Accumax (Innovative Cell Technologies) and replated onto Corning® Matrigel® Growth Factor Reduced (GFR) Basement Membrane Matrix-coated dishes in DFK 5% medium. NPCs were treated with TG003 (Sigma-Aldrich, 50 μM), SRPIN340 (50 μM) or Okadaic acid (FUJIFILM Wako Pure Chemical Corporation, 10 nM) for 3 h, then harvested, and RNA was isolated as described above. SRPIN340 was used as previously described [24].

### Recovery of neural phenotypes in SS-NPCs with TG003

For rescue experiments, we treated SS-NPCs isolated from day 12 cell aggregates with TG003 for 2 days before validating replication stress response and analyzing the mitotic spindle formation as described above.

### RNA-seq

RNA-seq analysis was performed at the Kazusa DNA Institute (Chiba, Japan). RNA-seq libraries were prepared by using a SureSelect Strand Specific RNA Library Preparation Kit (Agilent Technologies, Santa Clara, CA, USA). Sequencing was performed on an Illumina HiSeq 1500 (Illumina, San Diego, CA, USA) in 100-base pair-end mode. The mRNA profiles were expressed as fragments per kilobase of exon model per million mapped fragments. Principal Component Analysis (PCA) was performed with GeneSpring GX software (Agilent Technologies).

### GSEA

GSEA (http://software.broadinstitute.org/gsea/index.jsp) was used to estimate the molecular signatures [25, 26]. FPKM datasets were ranked for each analysis by using the Molecular Signatures Database (version 6.2; gene ontology (GO) gene sets, size filters set at 15-5000).

### GSE accession numbers

RNA-seq datasets were deposited in the GEO database and can be accessed with the GEO accession number GSE121384.

### Statistics

Statistical functions of GraphPad Prism6 (GraphPad Software, La Jolla, CA, USA) were used for the statistical analyses. Results are expressed as mean ± SEM. Statistical significance was determined using Student’s *t*-test unless otherwise described. *P* < 0.05 was considered significant.

## Results

### Establishment of SS-iPSC clones

**Fig. 1:**
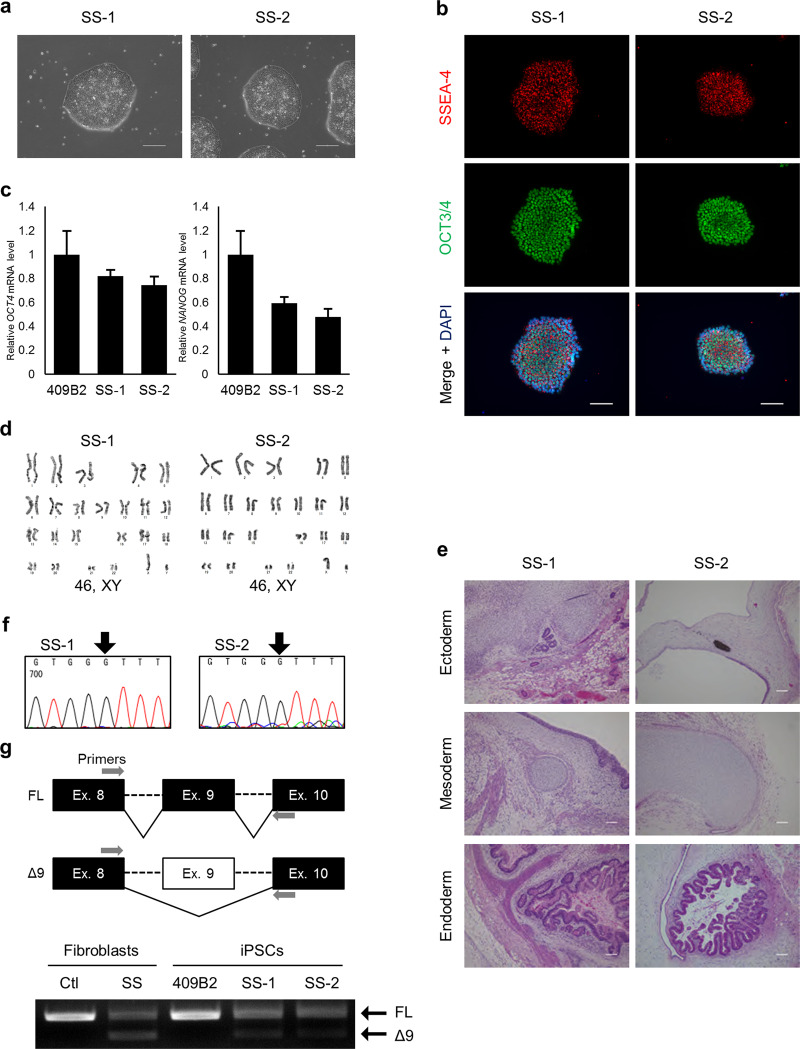
Characterization of SS-iPSCs. **a** Morphology of two SS-iPSC lines. **b** Immunostaining of pluripotent markers on SS-iPSCs. **c** G-banding and **d** relative RT-PCR analysis of PSC-associated genes. **e** Teratoma formation by the SS-iPSC clones. **f** Chromatogram results of Sanger sequencing ATR exon 9 show the existence of the *ATR* mutation (c.2101 A>G) in the two established SS-iPSC clones. Black arrows represent the position of the *ATR* mutation. **g** Splicing pattern validation of SS patient-derived fibroblasts and SS-iPSCs with RT-PCR products performed with primers spanning exon 9. Healthy donor fibroblasts and 409B2 clone were used as control. FL, full length isoform; Δ9, exon 9 skipped isoform. Scale bars, 100 μm

To model the neural phenotypes of SS, we first aimed to establish iPS clones from fibroblasts of a SS patient harboring the homozygous mutation in *ATR* gene (c.2101 A>G) [3]. Although the reprogramming efficiency was low, we succeeded in establishing two SS-iPSC clones. The established clones demonstrated ordinary embryonic stem cell-like morphology and pluripotent stem cell (PSC) markers (Fig. 1a–c). They also showed normal karyotype and could differentiate into three germ layers in the teratoma formation assay (Fig. 1d, e). Finally, both iPSC clones carried the identical mutation in the ATR gene (Fig. 1f).

### *ATR* (c.2101 A>G) mutation demonstrates cell type-specific splicing

Since fibroblasts carrying the *ATR* mutation show exon 9 skipping [3], we checked whether the aberrant splicing is maintained in iPSCs. Unexpectedly, when compared to fibroblasts in which exon 9-skipped (Δ9) isoform was predominantly transcribed, FL isoform was mainly expressed in iPSCs, and a small proportion of Δ9 isoform was expressed (Fig. 1g). These observations suggest that aberrant splicing of *ATR* exon 9 is a cell type-specific event and that there exist differences in the splicing machinery between these cell types.

**Fig. 2:**
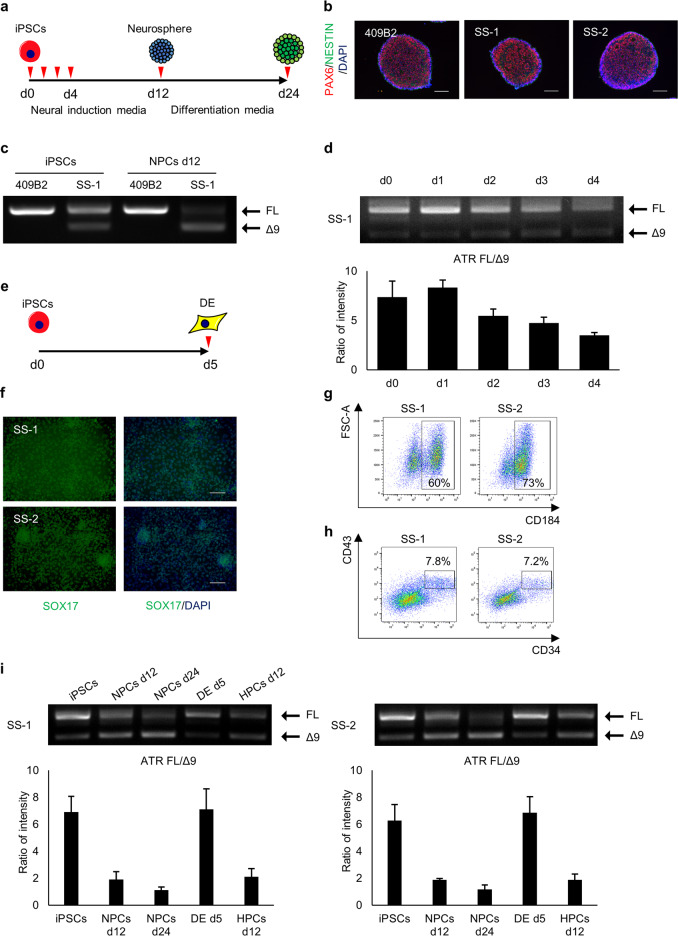
ATR c.2101 A>G mutation demonstrates cell type splicing. **a** Scheme of neural differentiation culture. Red arrows indicate the timing of splicing validation. **b** Immunostaining of SS-neurospheres at day 12. PAX6, red; NESTIN, green. 409B2 was used as the control iPSC line. **c** Splicing pattern validation of SS-iPSC-derived cells with RT-PCR products performed with primers spanning exon 9 (*n* = 3, independent experiments). FL, full length isoform; Δ9, exon 9 skipped isoform. **d** Evaluation of ATR splicing patterns during early stage neural differentiation (day 0 to day 4). Quantification analysis was performed with the intensity ratio of the FL band and Δ9 band. Data represent the mean ± SEM (*n* = 3, independent experiments). **e** Scheme of definitive endoderm (DE) differentiation. The red arrow indicates the time of splicing validation. **f** Immunostaining of SS-iPSC-derived DE cells at day 5. **g** Flow cytometric analysis of endodermal marker CXCR4 on SS-iPSC-derived DE cells. **h** Flow cytometric analysis of SS-iPSC-derived HPCs. **i** *ATR* RT-PCR analysis of different SS-iPSC-derived cell types with primers spanning exon 9. Quantification analysis was performed with the intensity ratio of the FL band and Δ9 band. Data represent the mean ± SEM (*n* = 3, independent experiments). d, days

To analyze neural specific impairments, we next differentiated SS-iPSCs into neural progenitor cells using the serum-free floating culture of embryoid body-like aggregates with quick reaggregation (SFEBq) method [19, 20] (Fig. 2a). At day 12, both SS iPSC clones showed comparable differentiation ability with control iPSCs, including a three-dimensional structure composed of PAX6-positive NPCs (Fig. 2b). Although SS-iPSC clones did not show any distinguished differentiation defects, they showed the same abnormal splicing of ATR observed in fibroblasts (Fig. 2c). The change in splicing pattern started within the first couple of days of neural differentiation (Fig. 2d), suggesting that splicing impairment occurs just after the cells lose their pluripotency.

To analyze whether alleviation of the missplicing is restricted to the PSC stage, we differentiated the iPSCs into definitive endoderm (DE) (Fig. 2e–g) and hematopoietic progenitor cells (HPCs) (Fig. 2h). Although SS-iPS-derived HPCs showed a similar splicing pattern to SS-iPSC-derived NPCs, SS-iPSC-derived DE cells did not demonstrate aberrant splicing (Fig. 2i), indicating that the aberrant splicing of *ATR* exon 9 is prominent in certain cell types, such as neuroectodermal lineage cells and hematopoietic cells, while differentiation towards other lineages avoids missplicing. The abnormal splicing in NPCs was maintained at a later neural differentiation period (day 24, Fig. 2i). Overall, *ATR* (c.2101 A>G) mutation caused different patterns of exon 9 splicing in a cell type-specific manner.

### Generation of isogenic mutation-corrected SS-iPSC clones

To investigate the pathological roles of the *ATR* mutation in SS-iPSC-derived neural progenitor cells (SS-NPCs), we established isogenic control iPSC clones. Correction of the *ATR* mutation was done by using the clustered regularly interspaced short palindromic repeats / CRISPR associated proteins 9 (CRISPR/Cas 9) system with a gRNA specifically recognizing the mutant allele (Supplemental Fig. 1). As a result, we obtained a mutation-corrected SS-iPSC (cSS-iPSC) clone, in which accurate substitution of the homozygous mutant sequence was confirmed by Sanger sequencing (Supplemental Fig. 2). We confirmed cSS-iPSCs recovered the splicing of ATR exon 9 in both iPSCs and NPCs, without any detectable Δ9 isoform band (Supplemental Fig. 3). These results verified that the *ATR* (c.2101 A>G) mutation was necessary and sufficient for the cell type-specific aberrant splicing.

### Transcriptome analysis of SS-iPSC-derived cells

**Fig. 3:**
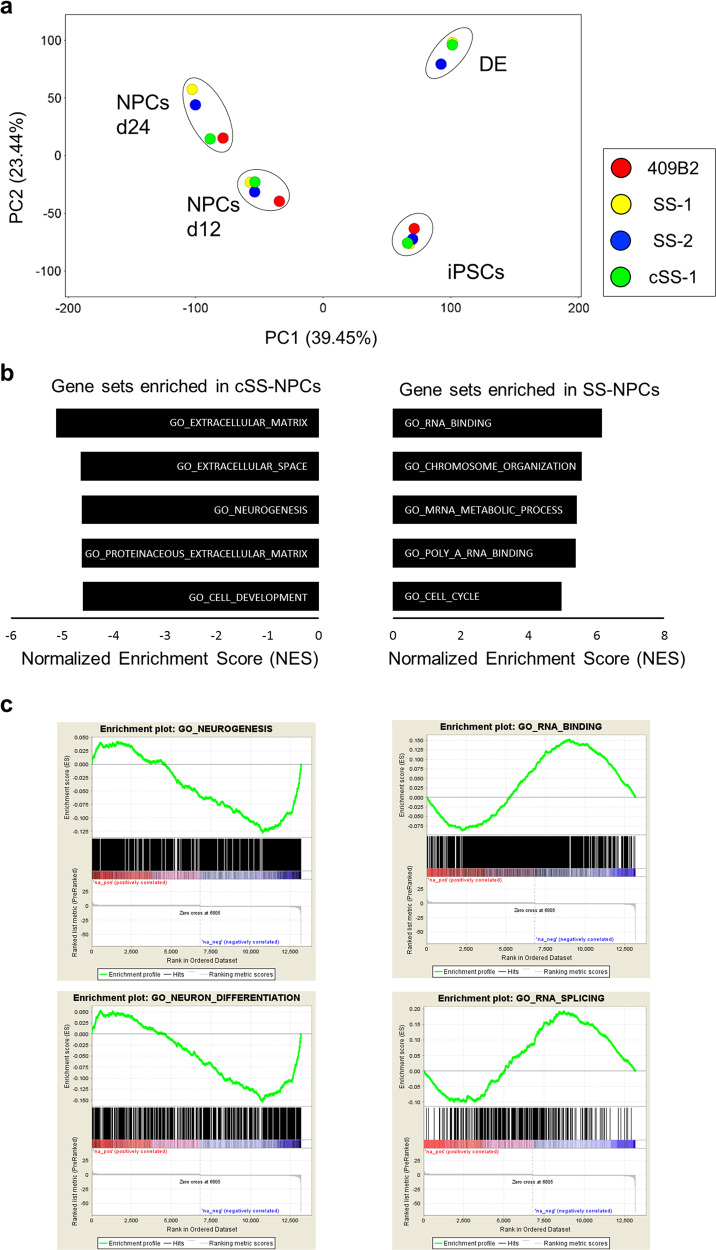
Transcriptome profile analysis of SS-iPSC-derived cells. **a** Principal component analysis based on RNA-seq data. Each colored dot indicates a cell line. Each cell type sample is surrounded with a black circle. **b** Normalized enrichment scores (NES) of the GSEA. The expression of whole genes between 2 SS-NPC clones and 1 cSS-NPC clone at day 12 was compared. Genes showing differential expression profiles were picked up and subjected to GSEA using gene ontology (GO) gene sets. The NES of the top 5 terms in the positive correlation gene set (SS-NPCs enriched gene set) and the negative correlation gene set (cSS-NPCs enriched gene set) are shown. **c** Representative GSEA plots from the top 20 terms. Left, Gene sets down-regulated in SS-NPCs. Right, Gene sets up-regulated in SS-NPCs

To characterize the global transcriptional effects of abnormal *ATR* splicing on SS-NPCs, we performed a RNA-seq analysis of iPSCs, NPCs, and DE samples derived from SS-iPSCs, cSS-iPSCs, and a control iPSC clone, 409B2. Principal component analysis (PCA) segregated the samples according to the stage of differentiation (Fig. 3a). When the iPSCs were differentiated into NPCs at day 24, ATR-mutated and wildtype clones located at separate positions, indicating ATR-mutant-specific transcriptional defects during neural differentiation (Fig. 3a). In order to identify the pathways associated with mutant ATR, we applied a gene set enrichment analysis (GSEA) on the expression of whole genes between two SS-NPC clones and one cSS-NPC clone at day 12. The SS-NPCs showed downregulation in gene sets related to extracellular matrix and neurogenesis compared with cSS-NPCs (Fig. 3b, c). In contrast, gene sets associated with mRNA processing including RNA splicing were upregulated in the SS-NPC population (Fig. 3b, c). Overall, these data indicated potential defects during the neuronal differentiation of SS-iPSCs.

### Impaired mitotic events and structural alignments of ATR-NPCs during neural differentiation

**Fig. 4:**
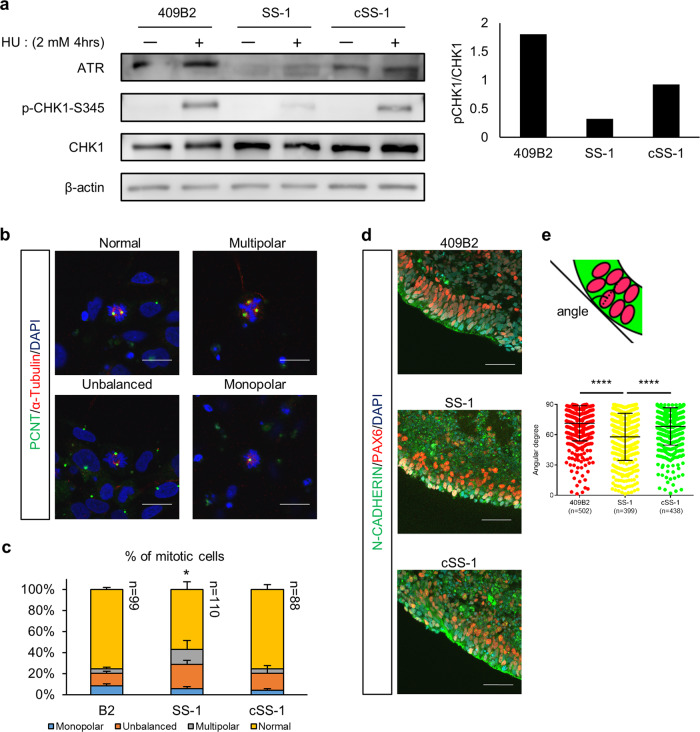
Neural defective phenotypes of SS-NPCs. **a** Western blot results of ATR activity against hydroxyurea (HU) in NPCs. Phospho-CHK1 (p-CHK1) and CHK1 were blotted, and the ratio of the p-CHK1/CHK1 blotting band was quantified. **b** Representative images of mitotic spindles in NPCs. Mitotic spindles were classified into four types (normal, multipolar, unbalanced, and unipolar). **c** Analysis of the mitotic spindle morphology in NPCs. Data represent the mean ± SEM (*n* = 2, independent experiments). **d** Immunostaining images of the outer layer of day 12 neurospheres. **e** Analysis of the nuclear position of NPCs within the surface layer of neurospheres. Data represent the mean ± SEM (*n* = 3, independent experiments; ****, *p* < 0.0001; one-way ANOVA test with multiple comparisons)

Because aberrant splicing occurred in SS-NPCs, the expression level of ATR protein was downregulated at this stage (Fig. 4a). Since a primary role of ATR is to initiate a cellular response to DNA replication stress, we evaluated the cellular response by treatment with hydroxyurea (HU), as reported previously [27]. In normal condition, HU treatment increased ATR-dependent phosphorylation of Checkpoint Kinase 1 (CHK1) protein [28], which initiates the DNA damage response. On the other hand, in SS-NPCs, phosphorylation of CHK1 was decreased, indicating an impaired response against DNA replication stress (Fig. 4a). cSS-NPCs recovered CHK1 phosphorylation to some extent, but not to the level seen in normal condition (Fig. 4a).

Since it has been reported that ATR stabilizes centrosomes and increases the centrosome number in ATR-defective cells [27, 29], and because mutations in a series of centrosome-associated genes were associated with SS [5, 6], we measured the mitotic spindle morphology of NPCs. Appropriate mitosis in NPCs is important, because it reflects proper development of the cerebral cortex [30]. We classified mitotic spindles morphologically into four types (normal, unbalanced, monopolar, multipolar), as described previously [21] (Fig. 4b). While normal mitotic spindles showed asymmetric alignment with two centrosomes, unbalanced spindles were characterized by an irregular alignment of microtubules or DNA or by the misplacement of centrosomes in the bipolar mitotic spindle. When plated NPCs derived from day 12 neurospheres were observed, impaired mitotic events, especially in multipolar spindle formation, were significantly increased in SS-NPCs (Fig. 4c). In cSS-NPCs, the frequency of abnormal mitotic events was recovered to the level in control iPSC-derived NPCs (Fig. 4c).

We also measured the alignment of NPCs at the outer layer of the neurospheres, because proper organization of NPCs in a uniform layer, such as the neuroepithelium layer, is necessary for proper neural development [31, 32]. In control neurospheres, the outer layer showed a clearly aligned group of cells expressing neural progenitor markers (Fig. 4d). In SS-iPSC-derived neurospheres (SS-neurospheres), the nuclei were aligned with high dispersion, a feature not observed in cSS-NPCs. To quantify the dispersed alignment, we measured the angle between a line tangent to the neurosphere and the long axis of the nuclei as an index of cellular alignment [33]. The angle in SS neurospheres and control clones was significantly different (Fig. 4e). Overall, these results suggest that, as was observed in the RNA-seq analysis, the ATR-deficient cell clone suffers from neuronal impairments.

### CLK1 inhibition promotes inclusion of mutated exon 9 in SS-NPCs

**Fig. 5:**
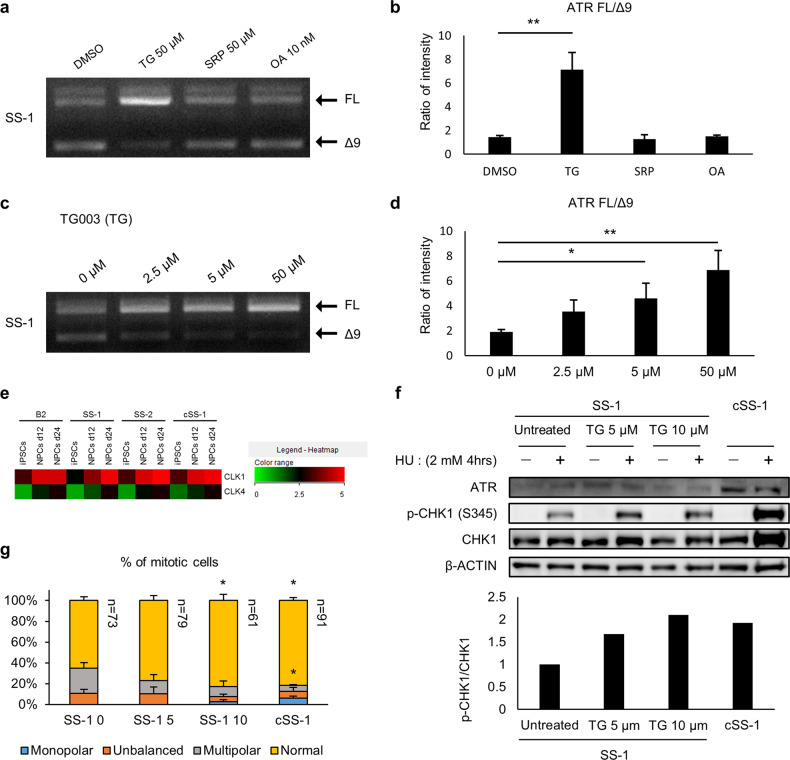
CLK1/4 inhibitor could rescue aberrant splicing of SS-NPCs. **a** Splicing pattern of *ATR* in SS-NPCs treated with SR protein modulators for 3 h. **b** Quantification analysis of the intensity ratio of the full length (FL) band and Δ9 band. Data represent the mean ± SEM (*n* = 3, independent experiments; *, *p* < 0.05; **, *p* < 0.01; one-way ANOVA test followed by Dunnett’s Multiple Comparison Test). **c** Dose-dependent effect of TG003 treatment on SS-NPCs for 3 h. **d** Quantification analysis of the intensity ratio of the FL band and Δ9 band. Data represent the mean ± SEM (*n* = 3 independent experiments; *, *p* < 0.05; **, *p* < 0.01; one-way ANOVA test followed by Dunnett’s Multiple Comparison Test). **e** Heatmap of the normalized expression value (log2FPKM) of CLK1/4 genes in 3 cell types of all clones. **f** Western blotting results of ATR activity against hydroxyurea (HU) in SS-NPCs after 2 days of TG003 treatment. p-CHK1 and CHK1 were blotted, and the ratio of the p-CHK1/CHK1 blotting band in HU-treated samples was quantified (bar graph). **g** Classification of mitotic spindles in SS-NPCs after 2 days of TG003 treatment. Data represent the mean ± SEM (*n* = 3, independent experiments; *, *p* < 0.05; two-way ANOVA test followed by Holm-Sidak’s Multiple Comparison Test)

In a previous study, the rescue of *ATR* splicing with antisense oligonucleotides or U1snRNA was verified in a minigene assay and ATR-SS model using mouse embryonic fibroblasts [34]. However, no pharmacological rescue for SS-related missplicing has been reported. Since the aberrant splicing of *ATR* is cell type-specific, differences in the activity of the splicing machinery, such as the status of serine and arginine-rich protein (SR protein) family members [35], might have a role. We, therefore, attempted to rescue this missplicing with chemical compounds that modify the splicing machinery. For this purpose, the effects of 3 splicing modifiers (TG003, SRPIN340, and Okadaic Acid), all of which regulate the status of SR proteins [24, 36, 37], were evaluated. TG003 and SRPIN340 inhibit SR protein activity by inhibiting different kinases (CLK1/4 and SRPK1/2, respectively) [24, 36], while Okadaic acid inhibits the activity of protein phosphatase 1, 2A and 2B to maintain the phosphorylation level of SR proteins [37]. Among these SR protein modifiers, TG003 exhibited a significant inclusion of exon 9, while the others had no effect (Fig. 5a, b). The effect of TG003 on exon 9 inclusion was dose dependent (Fig. 5c, d). Interestingly, CLK1 and CLK4 were upregulated in the neural lineages compared to iPSCs (Fig. 5e), suggesting that increased activity of these genes might be associated with the misplicing of mutated exon 9. Thus, treatment of SS-NPCs with TG003 increased the amount of FL mRNA of *ATR*. In line with this observation, the phosphorylation of CHK1 protein in SS-NPCs induced by DNA replication stress was recovered by TG003 treatment (Fig. 5f). Finally, we investigated whether TG003 could alleviate the abnormal mitosis observed in SS-NPCs. As a result, TG003 successfully increased the frequency of mitotic cells with mitotic spindles of normal morphology (Fig. 5g). Overall, TG003, a CLK1/4 inhibitor, could rescue the missplicing of *ATR* to alleviate the abnormal mitotic spindle formation in SS-NPCs. These results imply a possible involvement of SR proteins in the splicing of mutant *ATR* transcripts.

## Discussion

In this study, we established ATR-SS patient-derived iPSC lines to unveil the pathogenic mechanism in NPCs. There are several reports of PSC models of microcephaly other than ATR-SS [16, 38]. Because the development of NPCs is usually impaired in microcephaly and because NPCs are not directly obtainable from patients, PSC models are useful for understanding the pathophysiology. Although SS-iPSCs have also been established from a patient with *centrosomal P4.1-associated protein (CPAP)* mutation [16], to our knowledge, our study is the first to describe a PSC model of ATR-SS.

We found that abnormal mitotic events prevailed in SS-NPCs, which is a novel pathological feature of the disease. Along with governing the DNA repair pathway in the nucleus, ATR was reported to localize in the centrosome, indicating its potential role in controlling mitosis [39]. Indeed, abnormal centrosome formation was reported in patient-derived somatic cells [27, 29]. Our data provide direct evidence that the mitosis of iPSC-derived NPCs with the *ATR* mutation is also impaired, indicating the impaired structural organization of NPCs seen during brain development. In line with this observation, we confirmed that the alignment of NPCs in the outer layer of the sphere culture is somehow perturbed. Since alignment of the NPC nucleus so that it faces the apical surface indicates normal development of the neuroepithelium [33], these data support the idea that the ATR mutation affects both the mitosis and structural organization of NPCs.

Cell type-specific alternative splicing is a commonly used machinery to produce products with different functional properties [40], and approximately 95% of multiexon genes are considered to undergo alternative splicing [41]. In some genetic disorders, synonymous exonic or intronic mutations can cause alternative splicing of the gene, resulting in unexpected premature transcriptional termination and loss of protein translation [42]. Interestingly, PSC models have occasionally revealed that some of these mutation-associated aberrant splicings are cell type-specific [43]. Indeed, by using iPSCs and their differentiated progenies, we demonstrated here that the *ATR* mutation causes cell type-specific aberrant splicing. Although *ATR* (c.2101 A>G) mutation is the most investigated mutation among SS-related genes, there is no report describing the cell type-specific splicing associated with the mutation. Moreover, the insertion of the corresponding point mutation in mouse genomic *ATR* exon 9 could not induce skipping of the exon [44], indicating that this mutation could trigger cell type-specific splicing with human-specific machinery. Our study proved the usefulness of a PSC model to evaluate the spectrum of splicing patterns associated with potential disease-causing synonymous or intronic mutations.

Rescue of the aberrant splicing caused by the ATR mutation has been tried by using nucleic acid technologies such as ASO or modified U1snRNA [34]. Although these approaches showed clear rescue of the missplicing, there is no known pharmacological rescue using splicing modifiers. Moreover, the rescue of missplicing in neural lineage cells has not been reported, even though such investigations are important because of the different splicing machinery in different cell types. In this study, an in vitro trial of a small panel of splicing-modifying compounds identified TG003 as a reliever of the aberrant splicing. Moreover, the protein functions of ATR were recovered by TG003. Others groups have also demonstrated the possibility of TG003 rescuing abnormal splicing associated with other disorders such as Duchenne muscular dystrophy [45] or the inhibition of influenza A virus replication [46]. These findings including ours provide an in vitro proof-of-concept that splicing modifiers can recover the function and amount of the affected proteins. TG003 potently inhibits the activity of CLK kinases, which are SR proteins, affecting the alternative splicing patterns of various genes [47]. Therefore, differences in the activity of CLK kinases could be responsible for the mutation-associated aberrant splicing of ATR. However, to precisely understand the mechanism that causes the aberrant splicing, further studies including the evaluation of various SR proteins are required.

By establishing an isogenic mutation-corrected iPSC clone from SS-iPSCs, we could evaluate the effect of mutant ATR in NPCs precisely. Since *ATR* is a key regulator of the DNA repair pathway, it is expected that a loss of ATR function challenges genome editing methods. Apart from the advantage of acquiring the correct sequence of *ATR* exon 9, the alleviation of exon 9 missplicing at the PSC stage could facilitate the homologous recombination enhanced by the CRISPR/Cas9 system. The recovery of ATR protein function in PSCs may also be beneficial to obtaining the ATR-SS iPSC clones, which will advance models for other diseases with defective DNA repair pathways such as Fanconi anemia, since current reprogramming efficiency is extremely low [48–50].

In conclusion, our iPSC model revealed a novel function of the ATR mutation in NPCs and NPC-specific missplicing. Since PSC models of microcephaly with brain organoids are beneficial to dissecting the structural organization of NPCs during brain development, future work combining organoid technology with our ATR-SS iPSC model should be considered. Identification of the responsible splicing factors controlling the splicing pattern of ATR in both undifferentiated PSCs and NPCs would also contribute to understanding the function of splicing factors and the significance of alternative splicing during neural differentiation.

## Supplementary information

Supplementary materials
