# Supplementary material for: Verification and rectification of cell type-specific splicing of a Seckel syndrome-associated ATR mutation using iPS cell model
Source: J Hum Genet. 2019 Mar 8;64(5):445–58. doi: 10.1038/s10038-019-0574-8 (PMC8075875; doi:10.1038/s10038-019-0574-8)
Supplement: Supplementary file 1 — Supplementary materials [file 10038_2019_574_MOESM1_ESM.pdf]

Supplementary materials

Verification and rectification of cell type-specific splicing of a Seckel syndrome-associated ATR mutation using iPS cell model.

Jose Ichisima, Naoya M. Suzuki, Bumpei Samata, Tomonari Awaya, Jun Takahashi, Masatoshi Hagiwara, Tatsutoshi Nakahata, Megumu K. Saito

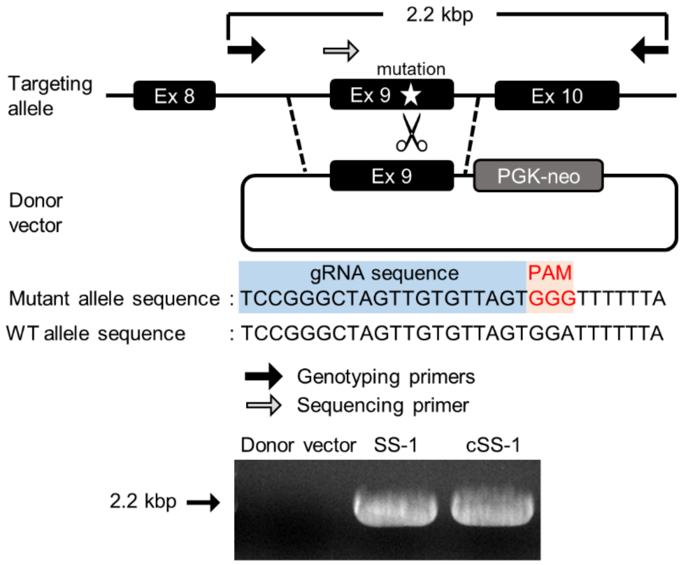

Supplemental Figure 1. Representative scheme of the gene editing strategy to establish cSS-iPSC clones. Black arrows indicate the genotyping primers, and white arrows indicate primers used for the Sanger sequencing. Position of designed gRNA sequence within mutant allele is shown. Bottom, representative RT-PCR result of genotyping PCR. WT, wild-type; PAM, protospacer adjacent motif.

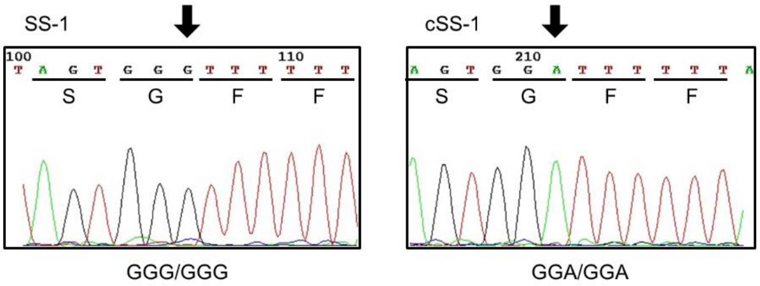

Supplemental Figure 2. Chromatogram results of Sanger sequencing *ATR* exon 9 show the correction of the *ATR* mutation (c.2101 A>G) in the cSS-iPSC clone. Black arrows represent the position of the *ATR* mutation.

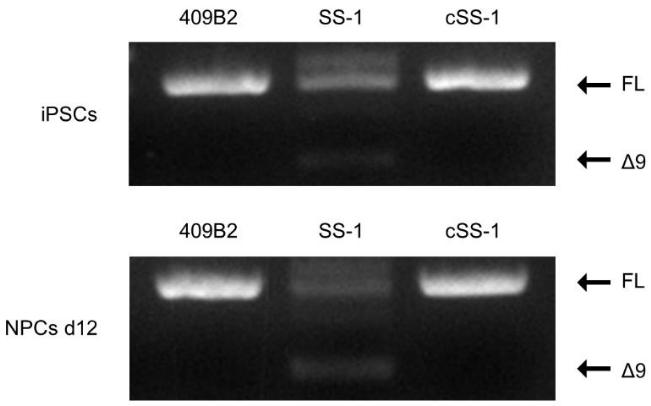

Supplemental Figure 3. The splicing pattern of cSS-iPSC-derived cells shows a complete loss of Δ9 isoform compared to SS-iPSC-derived cells.

Supplemental Table 1. Primer settings

Splicing validation primer	
ATR exon 9 val F	CCATTCTGATGATGGCTGTTT
ATR exon 9 val R	GTCCACATGTCCGTGTTCAG
Genotyping primers	
ATR-Crispr-validationF	GAAATTGTTTTGCTTCTTACAGCTT
ATR-Crispr-validationR	GGGAGTACTTTCTATTGCTTTCAA
Sequencing primer	
ATR A2101G F	AGCACCACAGGCACAATCAC
qPCR primers	
GAPDH qPCR-F	AATCCCATCACCATCTTCCA
GAPDH qPCR-R	TGGACTCCACGACGTACTCA
OCT4 qPCR-F	CTGGGTTGATCCTCGGACCT
OCT4 qPCR-R	CACAGAACTCATACGGCGGG
NANOG qPCR-F	AAAGAATCTTCACCTATGCC
NANOG qPCR-R	GAAGGAAGAGGAGAGACAGT
